# *Mycetohabitans rhizoxinica* bacteremia in the setting of invasive fungal disease in an immunocompromised patient

**DOI:** 10.1128/asmcr.00014-24

**Published:** 2025-04-02

**Authors:** Marisa Orbea, Mary Fortini, Megan H. Amerson-Brown, Debra L. Palazzi, James J. Dunn

**Affiliations:** 1 Department of Pediatrics, Infectious Diseases Section, Baylor College of Medicine and Texas Children’s Hospital 506057, Houston, Texas, USA; 2 Department of Pathology, Baylor College of Medicine and Texas Children’s Hospital 3989, Houston, Texas, USA; Pattern Bioscience, Austin, Texas, USA

**Keywords:** *Mycetohabitans rhizoxinica*, *Rhizopus*, *Exserohilum*, endofungal bacteria, sinonasal fungal disease, fungal pneumonia, invasive fungal disease, immunocompromised host

## Abstract

**Background:**

*Mycetohabitans* [*Burkholderia*] *rhizoxinica* is an endosymbiotic bacteria of *Rhizopus microsporus* that normally causes rice seedling blight. In our case report, we present one of the first known cases of concomitant bacteremia with *M. rhizoxinica* and invasive mold infection due to *Rhizopus* spp. in an immunocompromised child.

**Case Summary:**

A 3 year old male recently diagnosed with B-cell acute lymphoblastic leukemia developed febrile neutropenia. His workup was significant for invasive fungal sinusitis due to *Exserohilum* spp. based on histopathology and culture, a right middle lobe infiltrate, and a blood culture positive for gram-negative coccobacilli, later identified as *M. rhizoxinica*. Additionally, metagenomics next-generation sequencing was positive for *R. microsporus* as was broad-range fungal PCR testing of a lung biopsy sample. His surgical treatments included sinus debridement and a near total right pneumonectomy, and his antimicrobial treatment included 10 days of cefepime for his bacteremia, 4 weeks of liposomal amphotericin B, 4 weeks of micafungin following his pneumonectomy, and approximately 12 months of posaconazole.

**Conclusion:**

This case highlights the association of *M. rhizoxinica* with *Rhizopus* spp., wherein the isolation of *M. rhizoxinica* led to a high index of suspicion of *Rhizopus* infection in an immunocompromised patient who developed bacteremia with a slow-growing, oxidase positive, gram-negative bacteria not able to be identified by traditional identification methods.

## INTRODUCTION

*Rhizopus microsporus* is a ubiquitous environmental fungus that can cause invasive infections, including mucormycosis, in immunocompromised hosts. Previous ecological studies have identified strains of *Rhizopus microsporus* harboring endosymbiotic bacteria which produce rhizoxin, a potent antimitotic agent that causes rice seedling blight (1). Phylogenetic studies using 16S rRNA gene sequencing identified these bacterial organisms as two novel species, *Mycetohabitans* [*Burkholderia*] *rhizoxinica* and *Mycetohabitans* [*Burkholderia*] *endofungorum* (1). Strains of these bacteria isolated from eight adult patients were submitted to the Centers for Disease Control and Prevention between 1989 and 2006. Seven were obtained from blood specimens and one from wound tissue. No clinical background was provided, and contamination could not be entirely excluded as the source for the presence of the bacteria (2). As of this report, few known fungal infections have been documented in previous clinical cases of *M. rhizoxinica* bacteremia. The following unique case describes the occurrence of *M. rhizoxinica* bacteremia in a pediatric patient with concomitant invasive fungal infection caused by *Rhizopus microsporus*.

## CASE PRESENTATION

A 3-year-old previously healthy male presented with a 1-day history of fever and a swollen finger. A complete blood count demonstrated 59% blasts (reference range = 0), and he was diagnosed with B-cell acute lymphoblastic leukemia (B-ALL) as well as herpetic whitlow. On day 3 of hospitalization, he was started on induction chemotherapy (vincristine, dexamethasone, PEG-asparaginase, intrathecal methotrexate) while continuing intravenous acyclovir. Computed tomography (CT) of the chest was performed on hospital day 13, after 7 days of febrile neutropenia not responsive to cefepime, and demonstrated a right middle lobe infiltrate. Vancomycin and liposomal amphotericin B (5 mg/kg/day) were initiated on hospital day 14.

Eleven sets of blood cultures (BD BACTEC, Sparks, MD) were drawn over 13 days. One aerobic blood culture bottle, drawn on hospital day 12, had bacterial growth after 69 hours of incubation. The Gram stain showed gram-negative coccobacilli, and an aliquot of the blood culture was analyzed using the Accelerate Pheno system (Accelerate Diagnostics, Tucson, AZ) for identification of commonly isolated gram-negative rods but failed to give an organism identification.

**Fig 1:**
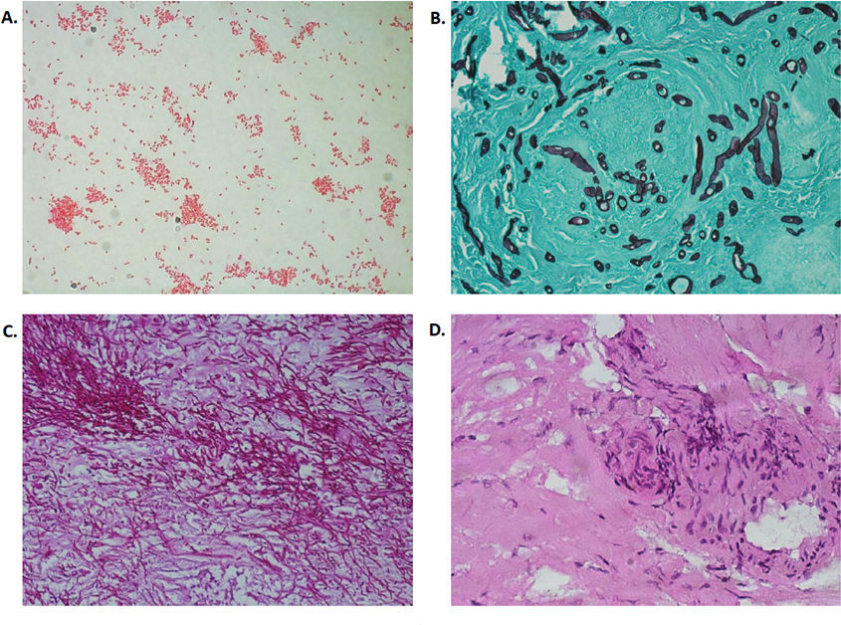
(A) Gram stain from the BAP shows gram-negative coccobacilli (100×). Representative images of FFPE left turbinate tissue sections stained with (B) GMS (40×), (C) PAS (40×), and (D) H&E (40×).

The organism grew on 5% sheep blood agar and chocolate agar within 24 hours of inoculation as grey round non-hemolytic colonies and was confirmed as gram-negative coccobacilli (Fig. 1A). There was no growth on MacConkey agar. The organism was oxidase positive, indole negative, and 3% catalase positive. No identification was given using matrix-assisted laser desorption/ionization time-of-flight mass spectrometry (Vitek MS, bioMerieux, Durham, NC) with the FDA-cleared IVD Vitek MS database version 3.2. Results of a clinically validated 16S rDNA gene pyrosequencing assay (sequencing of variable region 1, 3, and 6 of the 16 s rRNA) performed in the Texas Children’s molecular microbiology laboratory identified the organism as *Burkholderia rhizoxinica*, reclassified as *M. rhizoxinica* at the time of publication, with a 100% match in two curated sequence databases. Although Clinical and Laboratory Standards Institute guidelines do not provide break points for *M. rhizoxinica*, antimicrobial susceptibility testing was performed using ETEST (bioMerieux) on Mueller-Hinton agar (MHA) with 5% sheep blood, in 5% CO2 at 37℃ because the organism did not grow on MHA without sheep blood. Minimum inhibitory concentration values were ciprofloxacin, 0.5 µg/mL; ceftriaxone, 2 µg/mL; meropenem, 0.016 µg/mL; and cefepime, 1 µg/mL. Karius testing to identify microbial cell-free DNA (cfDNA) (3) was sent on hospital day 14 and identified *R. microsporus* at 14,826 molecules per microliter (MPM), Epstein-Barr virus at 60 MPM, and *Bacteroides vulgatus* at 58 MPM. The limit of detection for the Karius test is 10 MPM. Notably, Karius cannot detect *M. rhizoxinica* or molds like *Exserohilum* species. There was no evidence or clinical correlates suggestive of an ongoing infectious process driven by *Bacteroides*. For that reason, the patient did not receive therapy targeting Karius results other than *R. microsporus*. 1,3-beta-D-glucan and Galactomannan testing from serum samples were negative.

**Fig 2:**
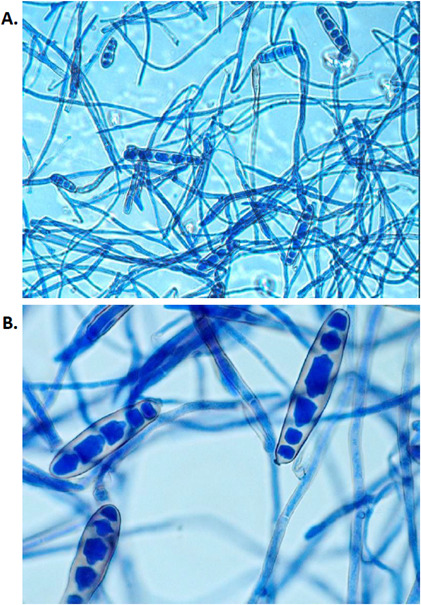
Lactophenol cotton blue stain of *Exserohilum* spp. from inhibitory mold agar (IMA) medium at (A) 40× and (B) 100× magnification.

CT scan of the patient’s sinuses showed non-enhancing mucosa around the left middle turbinate. Otolaryngology evaluation via nasal endoscopy revealed a necrotic eschar at that site. Left middle turbinate tissue was obtained during two separate surgical debridements and showed ribbon-like pauciseptate hyphae and thin septate hyphae with acute angles (Fig. 1B through D). The initial debridement yielded a floccus, dematiaceous mold. Microscopic examination of isolated colonies using a Lactophenol cotton blue stain (Fig. 2) revealed elongated and ellipsoidal conidia with distoseptate and strongly protruding, truncated hilum characteristic of molds in the genus *Exserohilum*.

**Fig 3:**
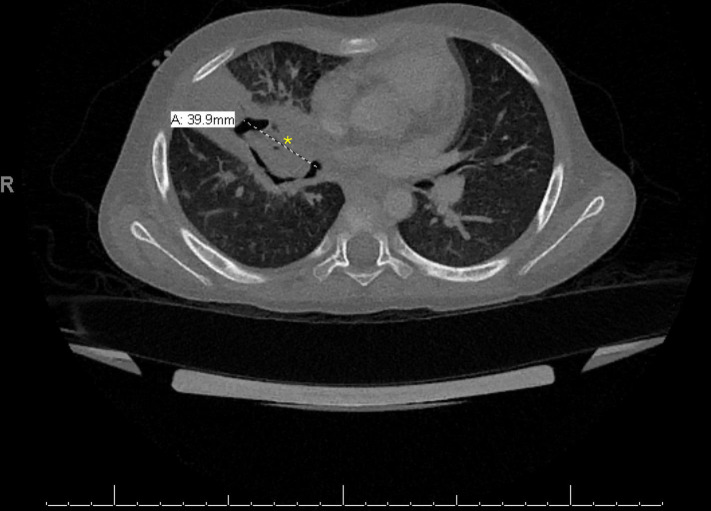
CT chest reveals a large cavity in the perihilar region with surrounding consolidation and a central soft tissue filling defect. Asterisk overlies cavity located in the right middle lobe, near the interlobar artery stump (approximately 7 mm).

Adjunctive therapy with posaconazole (10 mg/kg IV BID) and micafungin (3 mg/kg IV) once daily was added on hospital days 18 and 19, respectively, and liposomal amphotericin B dosing was increased to 10 mg/kg IV once daily. Separately, the patient completed a 10-day course of cefepime, 50 mg/kg IV every 8 hours, for *M. rhizoxinica* bacteremia. The patient subsequently developed worsening cough, and repeat chest CT findings were concerning for a large right middle lobe cavitary lesion at the site of a previously identified infiltrate (Fig. 3). Due to the proximity of the interlobar artery stump to the cavitary lesion, he underwent a right near total pneumonectomy. Histopathological exam of tissue obtained intraoperatively revealed fungal hyphae within the necrotic center of the hilar cavitary lesion. Similarly, fungal hyphae were identified in the pulmonary vein. Although no organisms were recovered in fungal cultures, *R. microsporus* DNA was detected in a lung tissue sample using the fungal PCR with reflex to NGS test performed at the University of Washington, which targets fungal 28S and internal transcribed spacer (ITS) DNA sequences via PCR, followed by amplicon sequencing (4).

This patient survived his infection and was treated with liposomal amphotericin B for approximately 4 weeks, micafungin for approximately 4 weeks following his pneumonectomy, and posaconazole (eventually transitioned from IV to oral formulation) for approximately 12 months.

## DISCUSSION

Fungal endosymbionts in the family Burkholderiaceae retain key genes that allow for an active bacterial invasion of fungal hyphae and a means to establish a functional symbiosis (1). *M. rhizoxinica* and *R. microsporus* have a symbiotic relationship in which fungi play host to bacteria to obtain virulence factors and induce fungal spore formation (1). *M. rhizoxinica* can be isolated, grown in pure culture, and reintroduced into a fungal host (1). This bacteria has also been associated with clinical specimens without the presence of a fungal host (2). Nucleic acid detection methods have been useful for identifying Mucormycosis from both direct specimens and as free circulating DNA from plasma in patients with invasive fungal disease (5, 6).

*R. microsporus* was identified in our patient from the blood using cfDNA metagenomic next-generation sequencing and from lung tissue by broad-range fungal PCR and sequencing. Karius testing did not detect *Exserohilum* spp. or *M. rhizoxinica*, as they are not detected by the assay (3); however, both organisms were identified by traditional culture. Despite only one organism being isolated from the turbinate tissue, two different hyphal morphologies were seen on the formalin-fixed paraffin-embedded tissue sections. These morphologies included ribbon-like structures resembling *Mucorales* and thin-segmented hyphae. The different hyphal structures could indicate either structural interference by antifungals or the presence of two truly distinct molds. *Exserohilum* spp. are an uncommon etiology of invasive fungal sinusitis. A review of *Exserohilum* infections from 1975 to 2012 noted that 27.2% of the infected individuals were immunosuppressed; of these patients, 72.8% had systemic involvement, and approximately half of all cases involved the sinuses (7). *M. rhizoxinica* was only successfully grown in one blood culture bottle despite a lingering mucormycosis. *In vivo* changes as a result of B-ALL disease process or treatment could have led to the release of *M. rhizoxinica* from *R. microsporus* resulting in the observed transient bacteremia. Additionally, 16S rDNA sequencing may lead to low-confidence species-level identification for *Burkholderia*. However, since the Vitek MS version 3.2 database contains a wide range of *Burkholderia* species, excluding *Burkholderia rhizoxinica*, and the corresponding bacterial host, *Rhizopus*, was identified in this patient, the likelihood of a missed identification on Vitek MS and a misidentification from 16S rDNA sequencing is low.

Effective treatment for both *Exserohilum* and *Rhizopus* spp. fungi involves early surgical excision/debridement and early initiation of antifungals (liposomal amphotericin B). Combination therapy with liposomal amphotericin B and posaconazole or liposomal amphotericin B and echinocandins has resulted in successful clinical outcomes; however, further evaluation of combination therapy is needed to prove efficacy (7).

To our knowledge, this is one of the first reports of *M. rhizoxinica* bacteremia in a patient with a confirmed concomitant invasive fungal infection, as determined by histology and laboratory diagnostic testing. The association of *M. rhizoxinica* with *Rhizopus* spp., even in the absence of direct isolation of *Rhizopus* spp., must be considered in at-risk patients, such as those who are immunocompromised. Identification of slow-growing, oxidase-positive, gram-negative bacteria by sequencing methods should be considered when the organism is not able to be identified by traditional methods. In conclusion, this unique case involving an immunocompromised pediatric patient highlights the need for additional studies to better understand the complex relationship between *Rhizopus* spp. and *M. rhizoxinica*, in particular, to identify *in vivo* conditions that trigger the release of endosymbiont bacteria from their fungal hosts.
